# A study of the solidification and stability mechanisms of heavy metals in electrolytic manganese slag-based glass-ceramics

**DOI:** 10.3389/fchem.2022.989087

**Published:** 2022-09-21

**Authors:** Jiaqi Wang, Fenglan Han, Baoguo Yang, Zhibing Xing, Tengteng Liu

**Affiliations:** ^1^ School of Material Science and Engineering, North Minzu University, Yinchuan, China; ^2^International Scientific & Technological Cooperation Base of Industrial Waste Recycling and Advanced Materials, Yinchuan, China; ^3^ Ningxia Institute of Geophysical and Geochemical Survey, Yinchuan, China; ^4^ Hubei Key Laboratory of Yangtze River Basin Environmental Aquatic Science, School of Environmental Studies, China University of Geosciences, Wuhan, China

**Keywords:** glass-ceramic, heavy metal contamination, curing/stabilization, electrolytic manganese slag, solid-waste management

## Abstract

To better solve the waste pollution problem generated by the electrolytic manganese industry, electrolytic manganese slag as the main raw material, chromium iron slag, and pure chemical reagents containing heavy metal elements mixed with electrolytic manganese slag doping. A parent glass was formed by melting the slag mixture at 1,250°C, which was, thereafter, heat-treated at 900°C to obtain the glass-ceramic. The results from characterizations showed that the heavy metal elements in the glass-ceramic system were well solidified and isolated, with a leakage concentration at a relatively low level. After crystallization, the curing rates of harmful heavy metals all exceed 99.9%. The mechanisms of heavy metal migration, transformation, and solidification/isolation in glass-ceramic curing bodies were investigated by using characterization methods such as chemical elemental morphological analysis, transmission electron microscopy, and electron microprobe. The most toxic Cr and Mn elements were found to be mainly kept in their residual state in the glass-ceramic system. It was concluded that the curing mechanism of the heavy metals in a glass-ceramic can either be explained by the chemical curing induced by bonding (or interaction) during phase formation, or by physical encapsulation. Characterization by using both Transmission electron microscopy and EPMA confirmed that Cr and Mn were mainly present in the newly formed spinel phase, while the diopside phase contained a small amount of Mn. Zn, Cd, and Pb are not found to be concentrated and uniformly dispersed in the system, which is speculated to be physical coating and curing.

## 1 Introduction

With the continuous expansion of the production of electrolytic manganese metals (EMMs), the waste residues, which are also produced in the process, are also increasing. The electrolytic manganese slag (EMS) comes predominantly from the following three processes in the electrolytic production of metal manganese: sulfuric acid leaking, oxidation iron removal, and pressure filtration removal of impurities (He et al., 2021; Zhou, 2021). With the existing EMMs production technology, every ton of produced manganese metal generates 10–12 tons of EMS (Ning et al., 2010). After decades of development, China alone has an inventory of over 100 million tons of EMS that, in the year 2020, has not been properly taken care of. And, with the continuous reduction of manganese ore resources in nature, the rate of new EMS production will exceed 10 million tons per year (He et al., 2021). Most of these manganese-containing waste residues are stored in dams and have not undergone any harmless form of treatment. Thus, they will not only occupy a large area of land resources but do also constitute a severe problem in terms of environmental safety. In the years 2010–2012, serious manganese slag dam failures and leaks did occur in Hunan, Sichuan, and Guizhou, which caused significant economic losses as well as environmental pollution. If the landfill of manganese slag is not in place to prevent seepage and major leakages, the manganese will seep into the soil and groundwater. This will cause serious problems in especially rainy areas. Coupled with the perennial weathering, the fine manganese slag particles will enter the atmosphere, becoming a great threat to environmental safety and human health (Röllin and Nogueira, 2011; Miah et al., 2020). The realization of harmless treatments and resource utilization of EMS is, therefore, a key step toward the sustainable development of the EMMs industry.

Many research groups around the world have performed a vast number of experimental studies in this direction. Shu et al. (2019) et al. compared the removal effects of hydroxide precipitation, sulfide precipitation, and carbonate precipitation on manganese and ammonia nitrogen in EMS. They found that the carbonate precipitation method is more efficient than the other two methods. For a C/Mn molar ratio of 1.1:1, and a pH value of 9.5 Mn was found to be excluded in the form of MnCO_3_, with a removal rate up to 99.9%. Furthermore, for a P/N molar ratio of 1.1:1, and a pH value of 9.5, ammonia nitrogen could be removed in the form of magnesium ammonium phosphate, with a removal rate as high as 97.4%. Xu et al. (2019) used a combination of EMS, blast furnace slag, and cement clinker to prepare cementitious materials. A mixture containing 15% EMS was shown to give the best material performance, with a flexural strength of 6.8 MPa, compressive strength of 32.9 MPa, and a 28 days strength of up to 52.5 MPa. And the heavy metals contained in the EMS in the system can be solidified well, and the levels of manganese and other heavy metals, as well as ammonia nitrogen, have reached the emission level of the national standard. However, the current methods cannot completely and effectively realize the harmless and resourceful treatment of EMS. This is mainly due to the disadvantages with a complex treatment process, excessive cost, low consumption of industrial waste residues, and unstable treatment of harmful elements.

In addition to electrolytic manganese slag, the production of solid waste chromium slag in the growing chromium salt industry causes serious pollution problems. The chromium slag composition is relatively complex. It is often composed of a variety of oxides, and accompanied by calcium chromate, magnesium chromate, and other carcinogenic substances. The chromium slag in addition to chromium, iron, magnesium and aluminum and other metal elements, but also contains highly toxic Cr (VI). The Cr (VI) ion in the chromium slag is characterized by a high content and high mobility. It can pollute soil, surface water, and groundwater, thus posing a threat to human beings and the surrounding ecological environment (Liu et al., 2020). A treatment of the chromium slag is, therefore, necessary to protect the environment and human health.

At present, the harmless treatment of chromium slag includes two categories; dry and wet methods (Zhang et al., 2013). However, wet detoxification of water and salt detoxification cannot effectively remove the acid-soluble hexavalent chromium ions. Furthermore, acid detoxification can be complete, but the acid dosage is large and there are high operating costs. There is also a problem with the outlet for the treated slag. Poor environmental safety stability of chromium slag after detoxification using alkaline methods. In addition, the wet detoxification method requires the addition of chemicals, and one must pay attention to not introduce new pollutants. The dry detoxification methods mainly use detoxification in the processes of cement making and iron sintering, as well as detoxification using a rotary kiln equipment. The detoxification in the process of cement making does mainly use a vertical kiln method, and this method is listed as a priority by the national industry restructuring. The usage has been reduced year by year, so this method is not a long-term alternative. The detoxification in the process of iron sintering is a complete method, and has a high potential for resource utilization. However, it is required that the treated chromium slag has a large quantity and a relatively stable composition. At the same time, there must also be available sintering iron-making equipment around the enterprise, otherwise there is a greater environmental risk problem in the process of long-distance transfer of chrome slag. In summary, the current harmless treatment of chrome slag has problems with an incomplete detoxification, high operating costs, and high environmental risks. In recent years, glass-ceramics have attracted more and more attention due to their excellent curing performance and good corrosion resistance, and are widely used in building materials, medical, industrial, and other fields (Zhao et al., 2021b). Chen et al. (2020) successfully synthesized high-calcium glass-ceramics by using ferromanganese-manganese slag as the main raw material and improving its crystallization by adjusting the content of Al_2_O_3_. The mass fraction of ferromanganese slag is 72.4%–88.7%. When the Al_2_O_3_ content is 11%, the flexural strength of the sample is 67 MPa and water absorption is 1.8%. Considering that there are a lot of SiO_2_, CaO, Al_2_O_3_, and MgO in the slag, the composition is like that of glass-ceramics. Slag containing MnO_2_, Fe_2_O_3_, and Cr_2_O_3_ can be used as an excellent nucleating agent. The large amount of energy consumed in the process of sulfur removal by high-temperature calcination of electrolytic manganese slag can be used as the condition of the melting stage. Based on the above analysis, the smelting process can be considered to prepare glass ceramics with electrolytic manganese slag as raw material and solidified heavy metal elements to achieve harmless treatment (Shu et al., 2020; Sun et al., 2020).

The harmless treatment of heavy metal elements in slags is not only depending on the presence and type of heavy metals in the system, but also their form, migration behavior, and solidification efficiency. The CMAS (CaO-MgO-Al_2_O_3_-SiO_2_) series of glass-ceramics have in the present study been successfully prepared from EMS. Furthermore, the degree of leakage, distribution of solidification, migration, and transformation of harmful heavy metal elements in glass-ceramics, have been studied and discussed. A comprehensive toxicity index model (STIM) was also established by calculating the potential toxicity of the various elements. It has generally been used to illustrate the ecological threat severity of heavy metals (Liu et al., 2008). In addition, the solidification mechanism of heavy metals has also been described in the present study. In addition, except for the pure chemical reagents used to simulate the composite heavy metal slag system, 100% of the slag is used as raw material, which saves cost and is in line with the concept of “treating waste with waste”. The finished glass ceramics have excellent performance, can meet the industrial use standard, and the leaching level of harmful heavy metals is also within the safe range. It can provide an effective way and choice for dealing with heavy metal pollution of solid waste.

## 2 Experimental

### 2.1 Sample preparations

The electrolytic manganese slag and ferrochrome slag that was used in the experiments in the present study were collected from an electrolytic manganese enterprise in China. The compositions of the two slag materials are presented in Table 1. The electrolytic manganese slag was subject to high-temperature calcination and de-sulfurization treatment, but the ferrochromium slag was not pre-treated. After both slags were retrieved from the enterprise, it was dried at 105°C for 1 h. It was, thereafter, crushed and ground into powder (after cooling) and passed through a 200-mesh sieve (with a particle size ≤75 μm). In addition to ferrochrome slag as well as electrolytic manganese slag, the chemicals used in the experiment include cadmium nitrate (CdN_2_O_6_.4H_2_O), lead oxide (PbO), hydrofluoric acid (HF), concentrated sulfuric acid (H_2_SO_4_), and concentrated nitric acid (HNO_3_) (Shanghai Hushi Laboratorial Equipment, China, CP), as well as zinc oxide (ZnO) and sodium hydroxide (NaOH) (Tianjin Kemiou Chemical Reagent, China, CP).

**TABLE 1:** Chemical composition of electrolytic manganese slag and chromite slag (wt%).

Material	SiO_2_	Al_2_O_3_	CaO	MgO	Fe_2_O_3_	Cr	Mn	S	Other
Ferrochrome slag	27.70	15.70	2.80	32.10	2.90	2.06	0.15	—	16.58
Electrolytic manganese slag	51.28	10.74	18.87	3.94	3.57	0.01	2.57	0.82	8.20

### 2.2 The preparation of glass-ceramics

Based on the results of XRF analysis, there were, with one exception, low concentrations of heavy metal elements in the electrolytic manganese slag. The ferrochrome slag was, therefore, selected for the preparation of glass-ceramics in the present study, in addition to pure chemicals containing Pb, Cd, and Zn. The latter ones were added to simulate a slag system containing a variety of heavy metals and blended with electrolytic manganese slag. The resulting material systems were used in the investigation of the effect of the glass-ceramic curing process on the curing of heavy metals in composite systems.

In the raw material systems, the ratio of electrolytic manganese slag to ferrochromium slag was 9:1, and the total mass of the mixed slag is regarded as 100%. The additional pure chemical added is calculated by the heavy metal monomeric elements contained therein and the corresponding mass of the chemical is weighed. The raw materials, with a total weight of about 50 g, were then mixed and stirred in a pot mill for 2 h.

Since the mass ratios of Cr and Mn were constant in the different samples, the addition of the three heavy metal elements (Pb, Cd, and Zn) could be used as experimental variables. The raw material samples are presented in Table 2.

**TABLE 2:** Amount of heavy metal elements added to each group of samples (wt%).

	Pb	Cd	Zn	Total
GC-1	1.0	1.0	1.0	3.0
GC-2	1.0	1.0	1.5	3.5
GC-3	1.0	1.5	1.0	3.5
GC-4	1.0	1.5	1.5	4.0
GC-5	1.5	1.0	1.0	3.5
GC-6	1.5	1.0	1.5	4.0
GC-7	1.5	1.5	1.0	4.0
GC-8	1.5	1.5	1.5	4.5

The raw material was, thereafter, placed in a corundum crucible and heated to above 1,250°C in a muffle furnace (KSL-1400X-A3, China). It was then kept for 150 min after which the material had melted into a glass liquid. The sample was, thereafter, immediately transferred to a preheated mold and kept in a muffle furnace for further annealing treatments. A parent glass was formed at the end of these annealing treatments (at specific holding times) and after cooling at room temperature.

**FIGURE 1:**
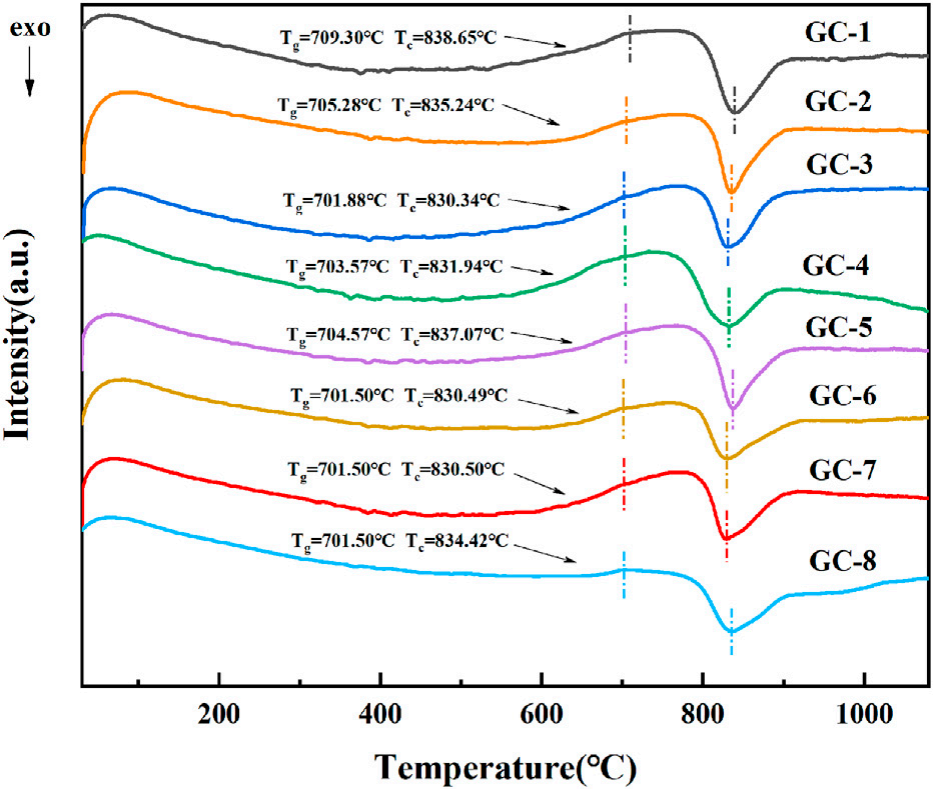
DSC spectra of different types of parent glass. (Exo: exothermic direction; T_g_: glass transition temperature; T_c_: crystallization temperatures; GC-1-8: Sample number).

A part of the parent glass was taken for grinding treatments, and the thereby formed glass powder was analyzed by using differential scanning calorimetry (DSC, STA 449 F3, Germany). When the raw material system reaches the transition temperatures (T_g_), it softens and absorbs heat, resulting in a slope in the endothermic direction on the curve. When the temperature reaches the crystallization temperatures (T_c_), crystals are precipitated in the system, and the arrangement of molecules changes from irregular to order. This process gives off heat and a clear exothermic peak appears on the curve. The heat treatment system could, based on these results, then be determined. The DSC results are shown in Figure 1.

The position of the crystallization peak in the DSC curve is about 830°C, and the heat treatment temperature is generally set at 50°C higher than the crystallization temperature. Therefore, the basic glass was held at 900°C for 150 min to complete the nucleation process, and the sample was cut and polished to obtain glass ceramics.

### 2.3 Sample characterization

X-ray diffraction (XRD; Shimadzu XRD 6000, Japan) was used to qualitatively analyze the phases in the samples. The following experimental parameters were used: working voltage = 40 k; working current = 30 mA; Cu-Kα ray; scanning rate = 2 /min: step size = 0.02°: 2θ Range = 10–80°. The chemical composition of the slag in the raw material was detected by using X-ray fluorescence spectroscopy (XRF, ZSX Primus II, Japan). A flat and regular glass-ceramic block was selected for the measurements. It was immersed in a 5% volume fraction of HF for more than 30 s and, thereafter, ultrasonically cleaned and dried. The morphology of the cleaned sample was analyzed by using an electron scanning microscope (SEM; Zeiss SIGMA 500, Germany). Moreover, the distribution of heavy metals in the glass-ceramic sample was investigated by using an electron probe microanalyzer (EPMA; Shimadzu EPMA-1720H, Japan) under the following experimental conditions: acceleration voltage = 15 kV; current = 50 nA; beam spot diameter = Min; step diameter = 0.1 μm; test time = 5 ms/point. The finely ground sample powder that should be analyzed was dispersed in ethanol, and the upper layer of the liquid (with the powder) was transferred to the sample holder and dried. Furthermore, a block sample was prepared by using FIB-SEM (Focused Ion Beam Scanning Electron Microscopy) by which it is possible to form a very thin test sample. Field emission transmission microscopy (TEM, JEM-2100, Japan) was, thereafter, used for the characterization of the microstructure of these very thin samples. An acceleration voltage of 200 kV, and a linear resolution less than, or equal to 0.14 nm were used for these measurements. Moreover, the binding energies of the electrons in the samples were characterized by using X-ray photoelectron spectroscopy (XPS; ESCALAB Xi+, United States ). In this analysis, the pass energy was 20 eV, the number of scans was 5, and the energy step size was 0.05 eV (since the adsorption of carbon introduces some errors in the measurements). Also, the XPS curve was fitted by using the binding energy of C1s (284.8 eV). With reference to the HJ/T 299-2007 Chinese environmental protection standard (HJ/T 299-2007, solid waste—extraction procedure for leaching toxicity—sulphuric acid and nitric acid method), the heavy metals in the glass-ceramics were extracted. In this extracted solution, the concentration of Cr(VI) was determined according to the Chinese national standard GB/T 15,555.4-1995 (GB/T 15,555.4-1995 Solid waste-Determination of chromium (VI)-1,5-Diphenylcarbohydrazide spectrophotometric method), and the concentrations of the other elements were measured by using ICP-MS(Inductively Coupled Plasma-Mass spectrometry). The heavy metal leaking rate, η, is expressed by Eq. 1 as a demonstration of the curing effect of the glass-ceramics.

$$\eta =C_{T}/C_{T0}$$ (1)

where C_T0_ is the total concentration of heavy metals in the system, and C_T_ is the concentration of heavy metals that have leaked from the sample.

The Synthesis Toxicity Index Model (STIM) can be used to evaluate the toxicity of the different heavy metals in the glass-ceramic curing systems. By using this model, the pH of the leakage in the BCR test has here been used to determine the values of bioavailability. The resulting values of bioavailability in the acid leaking state, reduction state, and oxidation state became 0.65, 0.4, and 0.2, respectively. A good environmental stability is needed for the residue state, and it is less likely to migrate than the states. Hence, bioavailability value for the residue state has been set to 0. See Table 3 for other specific parameter settings.

**TABLE 3:** STIM toxicity parameters and values.

Model parameters	Parameter value				
E_j_	E1 = 0.65	E2 = 0.4	E3 = 0.2		E4 = 0
Heavy metal	Zn	Pb	Cd	Mn	Cr
T_i_	1	5	30	1	2
Ci N (mg/kg)	58.8	20.6	0.11	524	60.6

The mathematical expression of the STIM is:

$$STIM=\sum_{i=1}^{n}[T_{i}\left(\sum_{j=1}^{m}E_{j}Q_{i}^{j}/C_{n}^{i}\right)]$$ (2)

where n is the number of heavy metal species, m is the number of the formed chemical species (m = 4), T_i_ is the toxicity response coefficient of the *i*th heavy metal (Hakanson, 1980; Xu et al., 2008), E_j_ is the chemical form j of the *i*th heavy metal bioavailability, Qj i is the content of the *i*th heavy metal chemical form j, and Ci N is the background value of the *i*th heavy metal in the natural environment. The background value of the local soil element in Ningxia has been used in this study (State Environmental Protection and China Environmental Monitoring, 1990).

The hardness of the glass-ceramic samples was tested by using a force of 5,000 g and a micro-Vickers hardness tester (432SVD, China). Furthermore, the flexural strength of glass-ceramic sample strips (of size 4 mm × 4 mm × 40 mm) was tested by using a universal testing machine (CMT5305, China). Measurements and calculation of the density, and water absorption of glass-ceramic samples are tested by Archimedes drainage. The acid and alkali resistance of the glass-ceramic samples was also evaluated by calculating the mass loss of the samples before and after immersion in 1% (v/v) H_2_SO_4_ and 1% (m/m) NaOH for 650 h, respectively. These tests were performed in triplicate to ensure the accuracy and reliability of the results. Calculated average values were used as the final data.

## 3 Results and discussion

### 3.1 Physical and chemical properties of glass-ceramics

**FIGURE 2:**
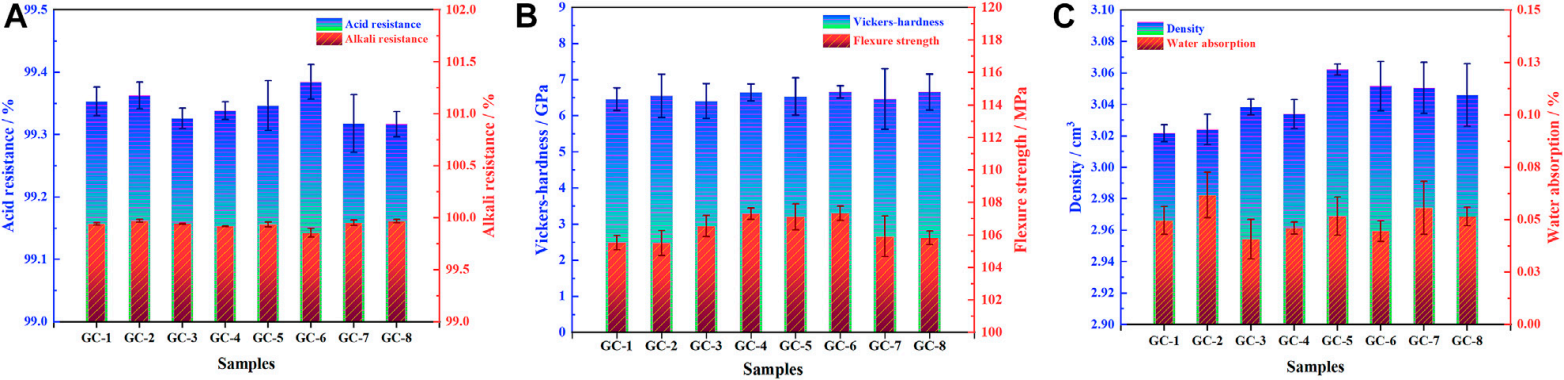
Physical and chemical properties of glass-ceramic solidified bodies. **(A)** Acid resistance and alkali resistance; **(B)** flexural strength and Vickers hardness; **(C)** density and water absorption rate.

After the final micro-crystallization of the parent glass, the stability of the cured bodies of harmful heavy metal elements has become closely related to the performance of the final glass-ceramic material. In the process of this crystallization, the generated crystalline phase has become intersected with the glass, forming a dense structure that can seal and isolate heavy metals and, thereby, play an encapsulating role. A higher density of the glass-ceramic system is correlated with improved cladding and improved stability of the heavy metal elements in the system. In addition, sintering occurs between the raw material powder particles during the preparation of the glass-ceramic, which makes the structure of the curing body denser and the surface flatter. The water absorption of the glass-ceramic can thus be kept at a low level. The reduction of water absorption will prevent harmful elements from leaking into the interior of the curing body, and make it difficult for corrosive liquids (such as acids and bases) to attack the system. This will, together with the excellent physical and mechanical properties of the curing system, ensure that the curing body material can still maintain its integrity when subjected to external forces. New exposure of surfaces, to the environment, can thereby be avoided, i.e., the interaction of heavy metals with the environment will be inhibited. In short, the better the physical and chemical properties of the curing system, the better its curing effect. The performance test results of each group of prepared samples are shown in Figure 2. However, the difference in performances, for each component, is not obvious. However, GC-6 show the best performance, with a density of 3.05 g/cm^3^, a water absorption rate of 0.04%, a flexural strength of 107.35 MPa, a Vickers hardness of 6.67 GPa, an acid resistance of 99.38%, and an alkali resistance of 99.86%. In fact, the overall performance of each glass-ceramic sample meets, and even exceeds, the requirements of China building materials industry standard (JC/T 2097-2011, glass - ceramics plate for industrial application).

### 3.2 Analysis of the glass-ceramic curing ability

#### 3.2.1 Analysis of toxicity leakage

The content of each element in the materials has been analyzed by using XRF. The experimental content of the two heavy metal elements, Cr and Mn, was found to be identical with the contents in the two kinds of slag materials: ferrochrome slag and electrolytic manganese slag. In addition, the content of Cd, Pb, and Zn in the original slag materials were negligible, so the addition of these elements in the glass-ceramic samples could be directly selected as the experimental content (as presented in Table 2).

The environmental damage caused by electrolytic manganese slag and ferrochromium slag can basically be attributed to the excessive content of Mn and Cr. The mass percentage of Mn in the electrolytic manganese slag was about 2.57%, and the mass percentage of Cr in the ferrochromium slag was 2.06%. The form of Cr that most easily diffuses into the environment is Cr (VI), which is carcinogenic. The content of Cr (VI) in the ferrochrome slag has, thus, severe toxicological effects on the environment. Therefore, the leaching and diffusion behavior of Cr (VI) has in the present study been given great attention in the toxicological leaching tests. However, the content of Cu, Co., Ni, Hg, and As in the mixed slag system was lower than the detection limit of ICP, so the present study did not consider the hazardous risks with these elements.

The toxicity leakage experiment was designed with reference to the HJT 299-2007 Chinese environmental protection standard, and Table 4 shows the concentration of heavy metals involved in the leakage experiment. The Cr (VI) element in the ferrochromium slag, and the Mn element in the electrolytic manganese slag, were 13 times and 430 times higher than the safe discharge standards, respectively. There were, thus, immense potential risks with these slags. After nucleation crystallization treatment, the leakage concentration level became much lower than the total elemental content in the feedstock system. The experimental results were compared with the allowed emission standard values (GB 5085.3-2007, Identification standards for hazardous wastes identification for extraction toxicity; GB 8978-1996, Integrated wastewater discharge standard). The results showed that the levels of all heavy metals were below the safety regulatory limits. This was especially the situation for the Mn and Cr (VI) levels, which were initially remarkably high in the original slag materials. This shows that by preparing a mixture of electrolytic manganese slag and ferrochrome slag, and transfer it into a glass-ceramic, the environmental toxicity of the heavy metals will be reduced very effectively.

**TABLE 4:** The leakage concentration of heavy metals in the toxicity leakage experiment (mg/L).

Samples	Cr (VI)	Cr	Pb	Cd	Zn	Mn
Ferrochrome slag	66.79	73.74	≤0.01	≤0.005	≤0.001	6.74
Electrolytic manganese slag	0.51	1.72	≤0.01	≤0.005	≤0.001	870.06
GC-1	≤0.01	0.01	0.96	0.88	0.45	1.64
GC-2	0.01	0.02	1.14	0.79	0.60	1.85
GC-3	0.01	0.02	0.68	0.57	0.83	1.20
GC-4	0.01	0.02	0.79	0.60	0.42	1.52
GC-5	≤0.01	0.01	0.79	0.93	0.59	1.55
GC-6	≤0.01	≤0.01	1.16	0.54	0.25	1.10
GC-7	0.01	0.02	1.05	0.60	0.74	1.60
GC-8	0.01	0.02	1.02	0.49	0.46	1.37
Toxicity threshold	5^a^	15^a^	5^a^	1^a^	100^a^	2^b^

a GB 5085.3-2007 Hazardous Waste Identification Standard Leaking Toxicity Identification.

b GB 8978-1996 Integrated Wastewater Discharge Standard.

**FIGURE 3:**
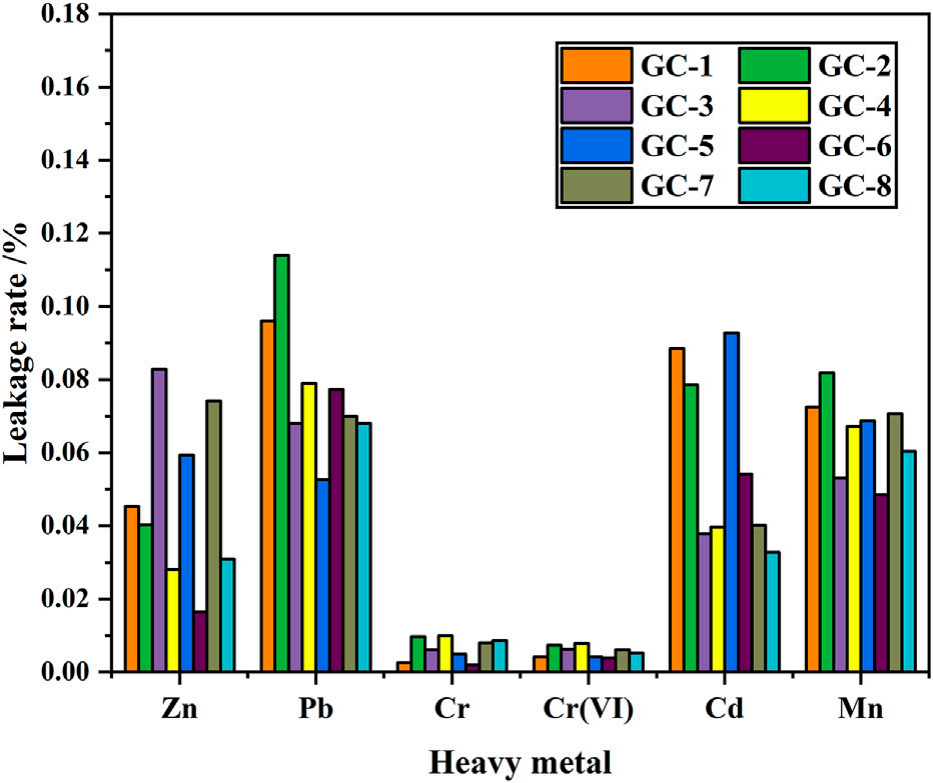
Leakage rate of heavy metals in glass-ceramic samples.

By comparing the leaching rates of several heavy metals, we have found that when the contents of the heavy metal elements are similar, the order of the solidification effect becomes: Cr (VI) > Mn > Zn > Cd > Pb, with solidification rates that all exceed 99.9%. Moreover, the curing efficiency of Cr (VI) was found to be the best, with the efficiency of larger than 99.99%. The leakage rate for each heavy metal element, in each curing system, is shown in Figure 3. It has been shown that the solidification in the glass-ceramics will effectively reduce the released amount of heavy metal elements. This is especially the situation for the environmentally most hazardous Mn and Cr (VI) elements. However, it should be noted that although the content of the other three heavy metal elements is lower than those of Cr and Mn, the level of solidification efficiency of Mn and Cr is higher. The reason is that both Mn and Cr act as nucleating agents during the preparation of the glass-ceramic samples. When the temperature is higher than the glass transition temperature, the Cr ions will attract the surrounding Fe, Mn, and Mg ions to form the spinel phase. Furthermore, when the temperature is increased to the crystallization temperature, the existence of the spinel phase can act as a heterogeneous nucleation core. It makes it easier to achieve the conditions required to produce a diopside phase, which precipitates and grows on the surface of the spinel phase. Thus, both Mn and Cr will more easily end up in the interior of the crystalline lattice and to become solidified and sealed (which also improves the microcrystalline structure to a certain extent). As a matter of fact, the crystallinity of the glass-ceramic will improve the solidification degree of also other heavy metal elements, as compared with the situation where only a single heavy metal element exists.

#### 3.2.2 Chemical analysis of heavy metal elements

**FIGURE 4:**
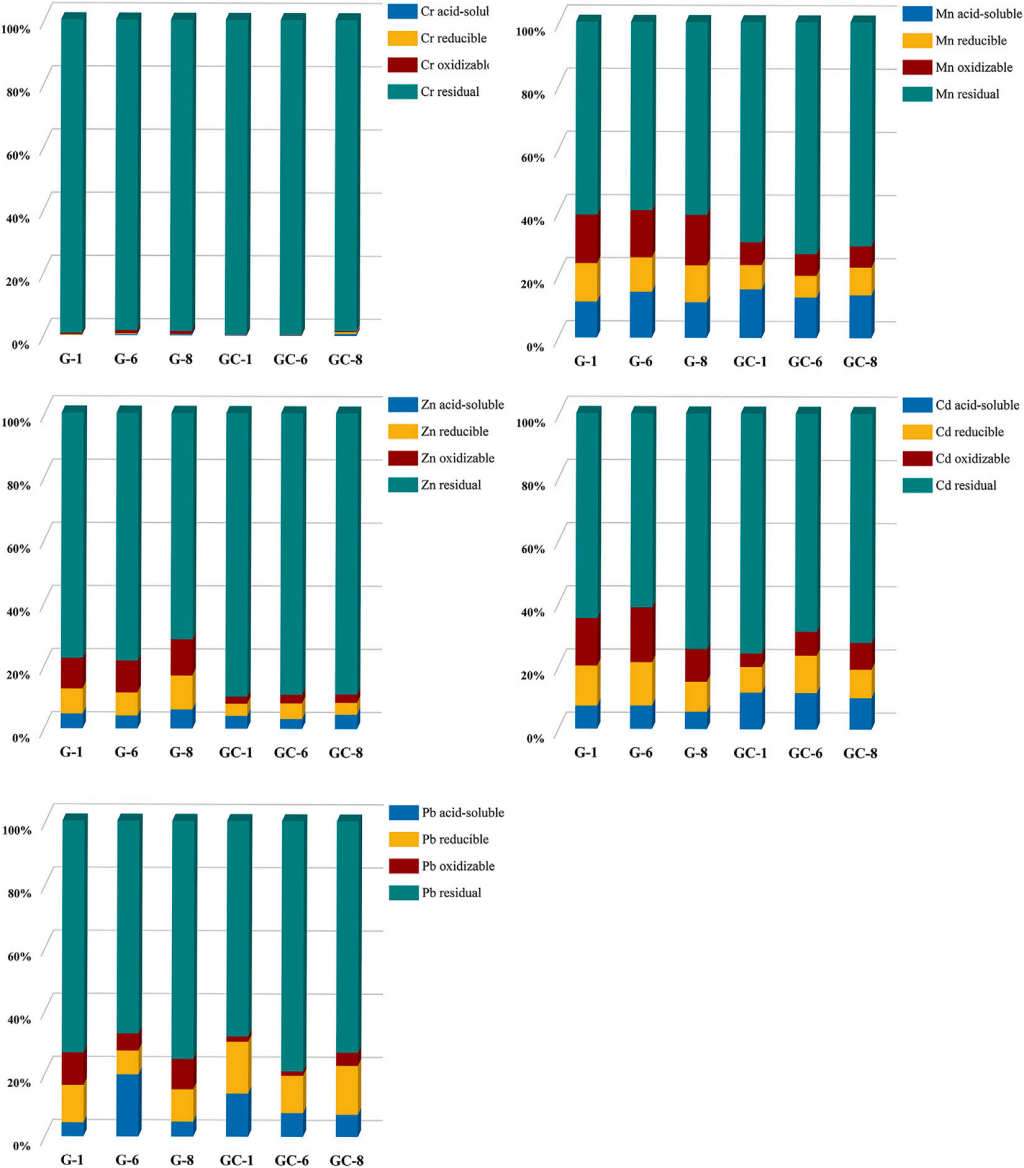
Distribution of Cr, Mn, Zn, Cd, and Pb elements in the parent glass and in glass-ceramic samples.

The curing stability of the heavy metals contained in the glass-ceramics and parent glass was found to be mainly related to the state of the heavy metal elements in the curing system and the curing bonding method. The experimental samples in groups 1 and 8 (i.e., with the lowest and highest additions of heavy metal elements) and the experimental sample in group 6 (with the best overall curing effect) have here been selected. The transient states of the five heavy metals in the parent glass, and in the glass-ceramic samples, were then tested by using the BCR (Community Bureau of Reference) extraction method. The glass-ceramic samples used for these tests were GC-1, GC-6, and GC-8, and the parent glass samples were G-1, G-6, and G-8. The BCR results are shown in Figure 4.

The heavy metal Cr element has been found to be predominantly in its residual state in both the glass-ceramic and in the solidified parent glass. The residual state of Cr in GC-1, GC-6, and GC-8 accounts for 99.49%, 98.59%, and 99.45%, respectively. Furthermore, it accounts for more than 98% in the parent glass samples. Moreover, the heavy metal Mn element was found to be predominantly in the form of its residual state and an oxidized state in the parent glass. The residual state accounts for about 60% and the oxidized state for about 15%. After heat treatment (i.e., after micro-crystallization), the percentage of oxidized state has become significantly reduced to a level of only about 7%, while the percentage of the residual state has increased to about 70%. Furthermore, the proportion of the heavy metal Cd element in its residual state has changed significantly, from about 65% in the parent glass to about 75% in the glass-ceramic. The proportion of the oxidized states in G-1,G-6, and G-8 became 15.10%, 17.35%, and 10.38%, respectively. Also, the proportion of the reduced states became 12.66%, 13.72%, and 9.47%, respectively. As a result of the heat treatment, these states became reduced to 4.34%, 7.56%, and 8.44% (oxidized state) and 8.08%, 11.88%, and 9.04% (reduced state). Furthermore, the heavy metal Zn element was found to exist in both the form of its residual state and the oxidation state in the solidified body of the parent glass. In the G-1, G-6 and G-8 samples, the percentage of the residual state was 77.67%, 78.48%, and 71.75%, respectively, and the percentage of the oxidation state was 9.71%, 10.13% and 11.43%, respectively. In the glass-ceramics GC-1, GC-6 and GC-8 samples (i.e., after heat treatment), the proportion of the residual state was observed to increase to about 90% for all three samples, and the proportion of the oxidized state was observed to decrease to 2.21%, 2.70%, and 2.69%, respectively. Furthermore, the oxidized state of the heavy metal Pb element was found to decrease from 10.37%, 9.59%, and 5.39%–1.64%, 4.19%, and 1.47% for the GC-1, GC-6, and GC-8 samples, respectively. In addition, the proportion of the Pb residual state in the glass-ceramic was also found to be higher than its level in the parent glass.

In a comparison of the two states (i.e., the residual state and the oxidized state) in the parent glass and in the glass-ceramic samples, the percentage of Cr, Mn, Cd, Pb, and Zn, in the residual state was observed to increase in glass-ceramic after heat treatment. The percentage of the more unstable acid leaked, oxidized and reduced states in the environment is significantly lower than that of the residual state, especially for the heavy metal Cr element. This is an indication on the fact that after crystallization treatment, the heavy metal elements do all change from the unstable state to the stable one. Thus, the stabilization of the heavy metals in the glass-ceramic has been further improved, as compared with the corresponding parent glass.

#### 3.2.3 Comprehensive toxicity assessment of the heavy metals

The ability of the heavy metal elements to migrate and transform in the environment, and their effects on the external conditions, has in the present study been examined by performing a leakage toxicity analysis and a chemical element analysis. However, the potential long-term toxicity effects by the heavy metals in the environment are not only related to the content of these elements, the activity of their chemical forms, and the level of leakage toxicity, but also to their chemical activity and bioavailability. A research group has previously established heavy metal toxicity assessment models by which it is possible to estimate the potential harm heavy metal elements can make to the environment (Zhang, 2016a). In the present study, STIM was been used to evaluate the degree of hazardous for each of the heavy metal elements in the glass-ceramic curing system (Luan et al., 2016).

**FIGURE 5:**
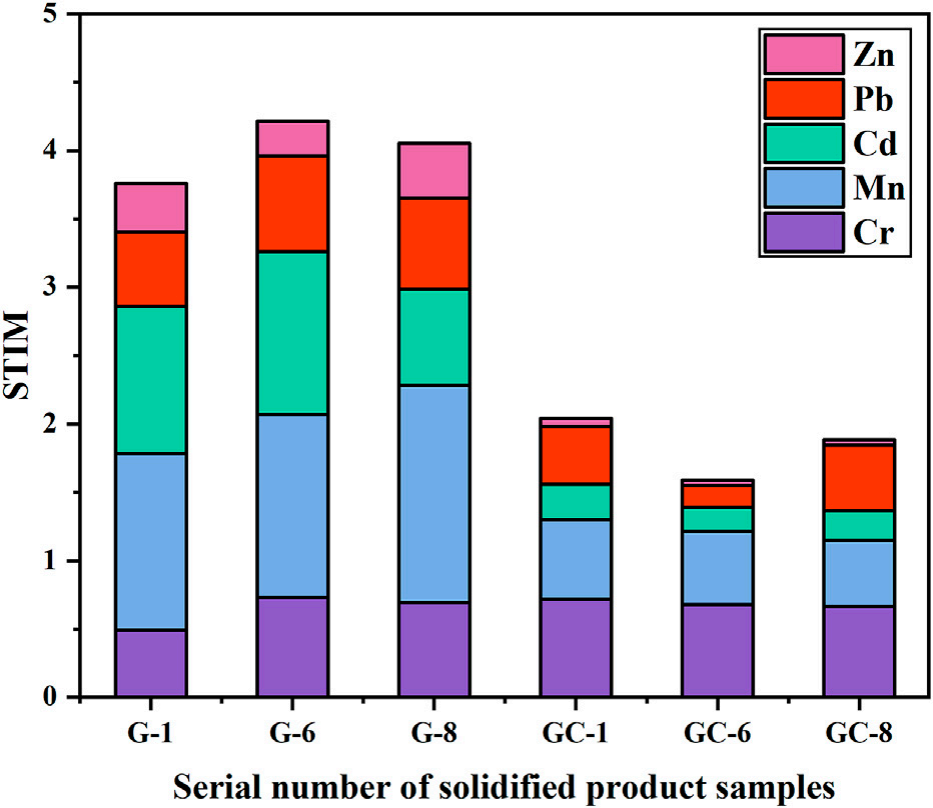
Comparison of STIM indices for heavy metal elements in the parent glass and in the glass-ceramic samples.

The results from the heavy metal toxicity analysis, for each group of glass-ceramic solidified bodies, can be seen in Figure 5. Eq. 2 has been used for these calculations.

The comprehensive toxicity indices of the parent glass samples G-1, G-6, and G-8 were found to have values between 3 and 4. Moreover, the STIM indices of the glass-ceramic samples GC-1, GC-6, and GC-8 were all found to be less than, or equal to, 2. Of all heavy metal elements studied in the present investigation, Cr and Mn are the main causes to environmental toxicity. As can be seen in Figure 5, the STIM indices of the glass-ceramic samples GC-1, GC-6, and GC-8 are all smaller than those of the corresponding parent glass samples (i.e., G-1, G-6, and G-8). Thus, the glass-ceramics have been found to effectively reduce the potential pollution risk of heavy metal elements in the ecological environment.

### 3.3 Heavy metal solidification mechanism

**FIGURE 6:**
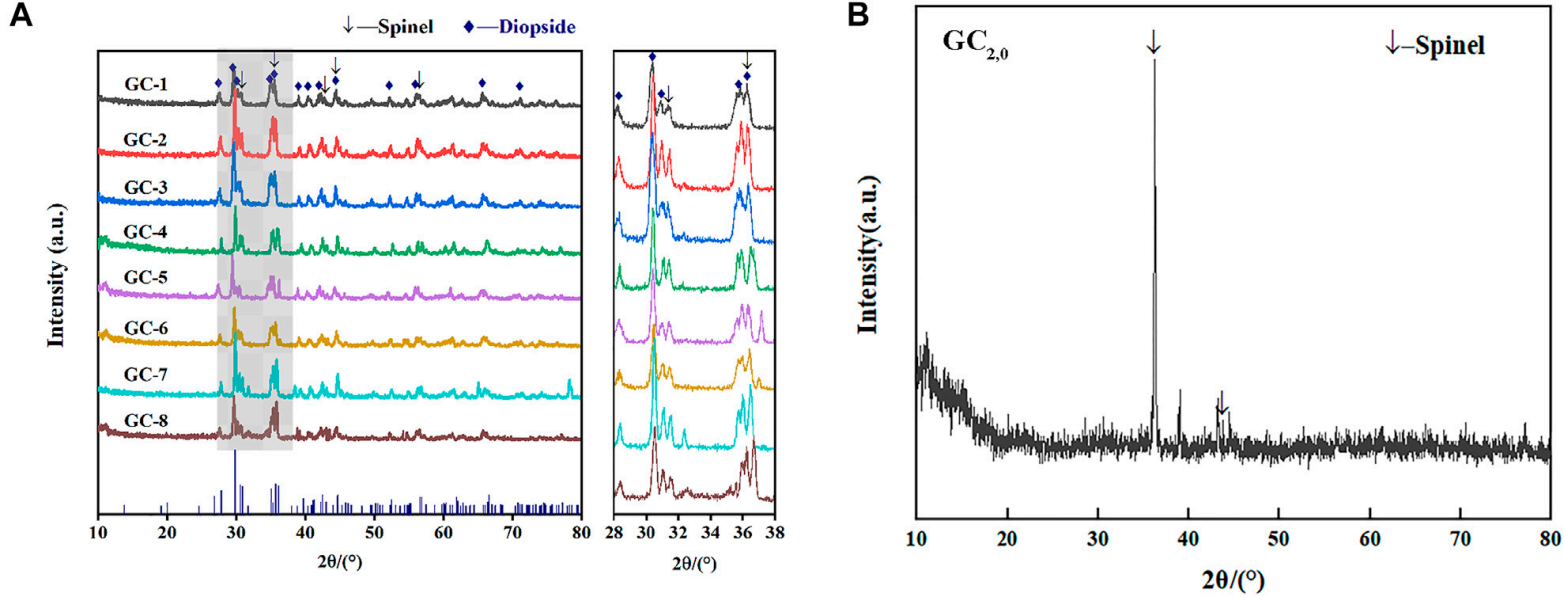
Comparison of XRD patterns for each group of glass-ceramic samples. **(A)** XRD patterns of GC1-8 samples; **(B)** XRD patterns of GC_2,0_ sample.

**FIGURE 7:**
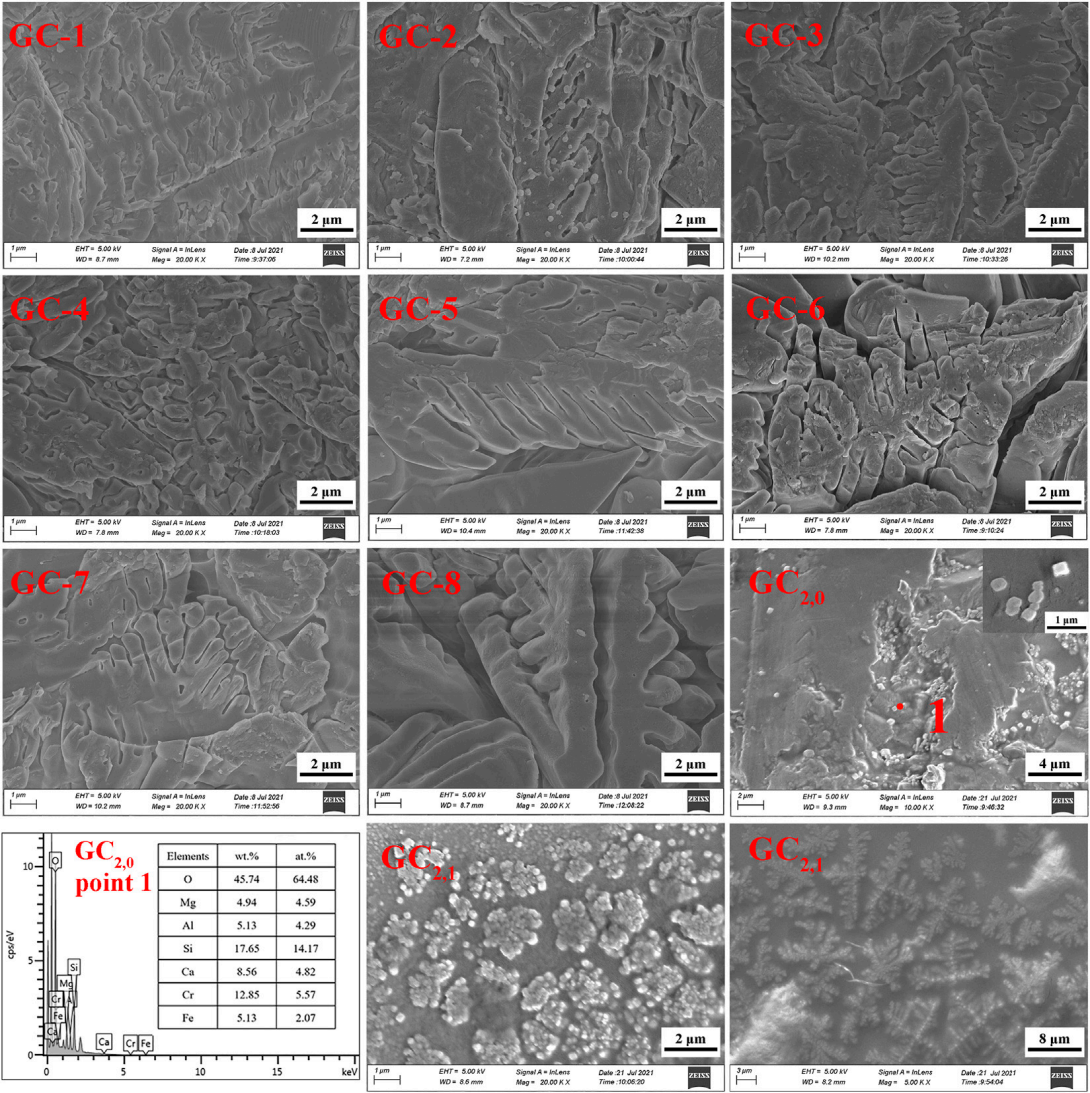
Comparison of SEM images for each group of glass-ceramic samples.

Figure 6A shows the XRD results for each glass-ceramic system (i.e., GC-1, GC-2, GC-3, GC-4, GC-5, GC-6, GC-7, and GC-8). It is obvious that all systems contain both diopside and spinel crystalline phases. The main crystalline diopside phase, tremolite, has a dendritic structure with a width of 1–2 μm (see the SEM images in Figure 7). Furthermore, it has been speculated that the generation of the spinel phase is due to the presence of Cr_2_O_3_ and Fe_2_O_3_ in the systems. The “ferrophilicity” and “oxyphilicity” exhibited by Cr^3+^, and the large field strength induced by the ion itself, will easily interact with the surrounding Mg and Fe plasma agglomerates. Therefore, in the nucleation stage, were the melted system reaches the glass transition temperature, the elements dominated by Cr, Fe, Mn, and Mg have produced inhomogeneous enrichment areas and, thereby, formed spinel phases (Zhao et al., 2019a; Zhao et al., 2019b).

The GC-0 group was added to the group of glass-ceramics to verify the cause of spinel production. The ratios of raw materials in this specific group were identical to those of the CG-6 group (which showed the best curing effect on the heavy metals). As was the situation with the other groups in the present study, the uniformly mixed slag material was annealed until it melted. According to the DSC test results, the nucleation and crystallization temperatures of GC-0 were 700°C and 830°C, respectively. For the nucleation of GC-0, the temperature was kept fixed at 720°C for 2 min. The hereby obtained nucleated glass was named GC_2,0_, and an XRD analysis was performed at this specific nucleation stage (see Figure 6B). It was found that after a short period of nucleation, only the spinel phase appeared in the system. This is a strong indication on the fact that the process of spinel generation started already in the nucleation stage.

The GC_2,0_ material was also analyzed by using SEM and EDS. A small-scale structure, in the form of an octahedron, can be seen in the SEM image in Figure 7. This is most probably the spinel phase that has been generated in the system. Moreover, the EDS results indicate that the material phase contains Cr and Fe elements and has a low Ca content (see Figure 7). This result is also consistent with the spinel characteristics.

GC_2,0_ was further subject to crystallization treatment at 900°C for 1 min, and the obtained crystallized glass was named GC_2,1_. As can be seen in the SEM images of GC_2,1_ in Figure 7, the spinel nuclei have agglomerated and has developed into a typical dendritic pyroxene crystalline structure. This observation gives evidence to the conclusion that the spinel phase has served as a heterogeneous nucleus for the main crystalline phase, tremolite. In other words, the generation of tremolite was induced by the spinel structure (Shi et al., 2018; Ayala Valderrama et al., 2019).

**FIGURE 8:**
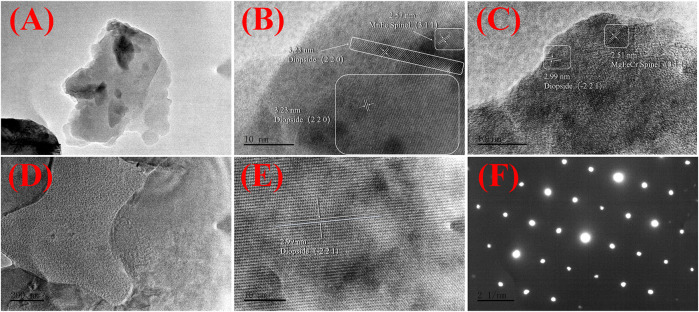
Transmission electron microscopy (TEM) characterization of the GC-6 sample: **(A)** A low-resolution TEM image of the GC-6 powder; **(B,C)** High-resolution TEM images of the GC-6 powder; **(D)** A low-resolution TEM image of the FIB-SEM ion thinning GC-6 sample; **(E)** A high-resolution TEM image of the FIB-SEM ion thinning GC-6 sample; **(F)** A SAED image of the FIB-SEM ion thinning GC-6 sample.

Moreover, Figure 8 shows low- and high-resolution TEM images of the GC-6 sample. Figures 8A–C are images of glass-ceramic powder samples that have passed through a 200-mesh sieve, and Figures 8D,E, and f are images of the FIB-SEM ion thinning samples. The observation and study of the microstructure revealed that the crystalline phase in the system is distributed a small-sized structures that are interleaved with the glass phase. Distinct regions of lattice diffraction streaks can be observed in the high-resolution TEM images in Figures 8B,C. These streaks are not uniquely oriented, with a small amount of an amorphous phase surrounding the region where the crystalline phase region. As demonstrated in Figures 8D,E, the samples were also analyzed by using high-resolution TEM on the ion-thinned samples. Uniform and regular crystal diffraction streaks were then observed, with no obvious amorphous regions. Moreover, only regular diffraction spots appeared in the SAED plot shown in Figure 8F, with no continuous diffraction rings. This indicates that the proportion of the crystalline phase in the glass-ceramic sample is much higher than the proportion of an amorphous phase. The diffraction stripe spacing in the regions shown in Figures 8B,C,E has also been analyzed by using the GMS 3 software. It was possible to find crystal regions with a diffraction stripe spacing of 0.299 nm and 0.323 nm that belong to the (−2 2 1) and (2 2 0) crystal planes of perovskite (Ca_1.022_(Mg_0.857_Fe_0.122_) ((Si_1.877_Fe_0.144_) O_6_), PDF # 89-0834), respectively. Moreover, the crystalline facets with 0.251 nm and 0.257 nm stripe spacing were found to belong to the (3 1 1) crystalline facets of spinel (MgFe_0.9_Cr_1.1_O_4_ PDF # 71-1255), and ((Mn_0.113_Fe_0.977_Ti_0.91_) [(Ti_0.09_Fe_0.815_Mn_0.095_) O_4_) PDF # 82-1293], respectively. It was then confirmed that most of the crystalline phases in the glass-ceramic sample are diopside, in addition to a smaller amount of spinel. In the curing system, heavy metal elements Cr, Mn is mostly atomic substitution, chemical bond binding or solid solution form in spinel phase. After the heat treatment crystallization process, the spinel phase further develops into the diopside phase to form a more stable curing system. Compared with the results of the XRD measurements, the conclusions that can be drawn from the TEM analysis further refer to comparison of lattice parameters, more accurate and effective.

**FIGURE 9:**
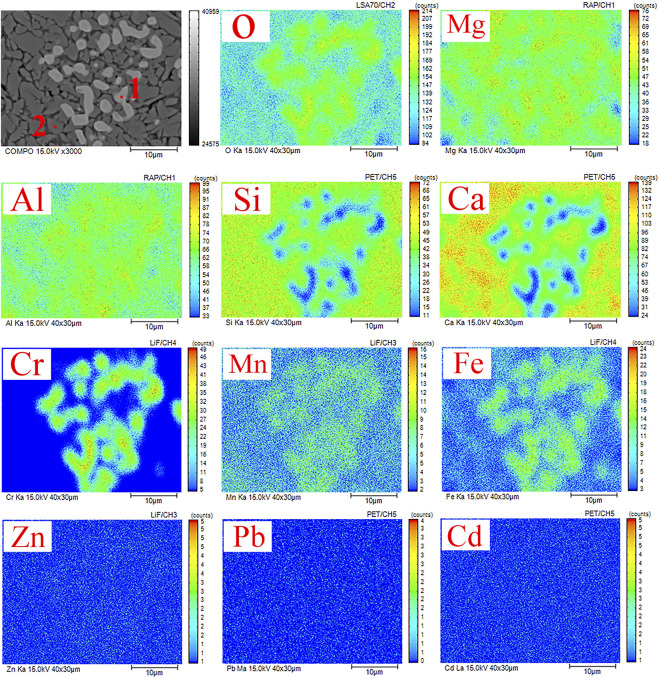
EPMA images of the GC-6 sample with its elemental distribution of O, Si, Ca, Mg, Al, Cr, Mn, Fe, Pb, Zn, and Cd.

Figure 9 shows the results of the qualitative and semi-quantitative analysis of the crystal phase region of the GC-6 glass-ceramic sample. Electron probe microscopy was used in this analysis, by which the distribution of each element in the micro-phase of the glass-ceramic phase could be obtained. The results from the energy spectrum analysis of points 1 and 2 in Figure 9 are shown in Table 5. The results show that the presence of the heavy metal elements Cr, Mn, and Fe became enriched in the spinel phase. Moreover, the dominating elements were O, Ca, Al, Si, Mg, Fe, Cr, and Mn. The contents of Cr and Mn were 13.52 and 22.42 wt%, respectively (see Figure 9, point 1). Due to the large field strength of the Cr^3+^ ions, and the “ferrophilicity” exhibited by the chromium oxides, the Fe, Mn, and Cr elements could together form a spinel phase which functioned as a heterogeneous nucleation core (Liao et al., 2016; Zhao et al., 2021a). Furthermore, the dominating elements in the diopside phase (see Figure 9, point 2), were O, Ca, Al, Si, Mg, Fe, Mn, and Cr. More specifically, the contents of Cr and Mn were 1.15 and 4.52 wt%, respectively.

**TABLE 5:** Results from the EDS analysis of points 2 and 3 in Figure 9.

Elements	1	2
O	32.18	46.06
Mg	3.22	5.62
Al	5.49	5.16
Si	10.89	20.64
Ca	5.94	12.64
Cr	13.52	1.15
Mn	22.42	4.52
Fe	6.33	4.21

The distribution of heavy metal elements in GC-6 is also shown in Figure 9. Combined with the TEM results, two elements, Cr and Mn, were found to be enriched in the crystalline phase that consisted of mainly spinel MgFe_0.9_Cr_1.1_O_4_ and (Mn_0.113_Fe_0.977_Ti_0.91_) [(Ti_0.09_Fe_0.815_Mn_0.095_) O_4_]. This is an important indication that both Cr and Mn can be effectively solidified by participating in the crystalline phase formation. Combining the results of part 3.2.2, most of the chromium and manganese elements were found to be in the residual state with a good curing effect. It can be concluded that both spinel (which forms a stable crystalline phase) and tremolite (which is formed onto the spinel core) possess an excellent resistance to leakage, making it difficult for heavy metals to migrate. Thus, the escape of harmful elements into the environment has been prevented. Whether heavy metal elements can exist for a long time in the solidified body mainly depends on their chemical form in the system, in addition to the way of solidification and bonding. The way that heavy metals form compounds can facilitate the replacement of heavy metals with elements forming crystal lattice in the process of phase transition (so as to enter the crystal and become solidified) (Zhang et al., 2016b).

Both Cr and Mn became solidified in the crystalline phase of the glass-ceramics, which significantly reduced the leakage toxicity of both elements. This result is consistent with the results in a previous study that showed that the leakage rate of heavy metals in the spinel phase is significantly higher than the leakage rate of heavy metal oxides (Su et al., 2018). Thus, the glass-ceramic solidified body was shown to have an excellent effect on the solidification and stabilization of heavy metals. The remaining three heavy metal elements (Zn, Cd, and Pb) were not concentrated in the crystalline phase but were dispersed and distributed in both the glass phase and in the crystalline phase. The reason may be that these heavy metal elements do not participate in the crystalline phase precipitation process, and the content is also low. They could not either precipitate into an independent phase, so these three elements became wrapped and isolated by a grid formed by the interlacing of crystalline and glass phases. Thus, the curing method for these heavy metal elements is physical wrapping.

**FIGURE 10:**
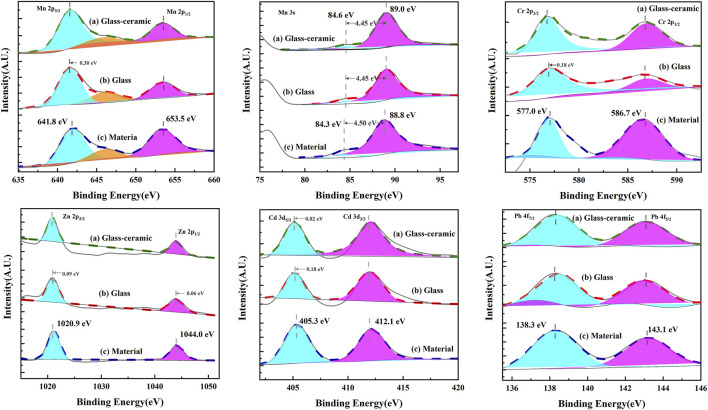
XPS spectra of heavy metals in the GC-6 sample.

X-ray photoelectron spectroscopy was also used in studying the electron binding energies of five heavy metal elements in the glass-ceramic samples of the GC-6 group, the parent glass, and a mixture thereof. Figure 10 shows the XPS spectra of Cr, Cd, Pb, Zn, and Mn. The C 1s peak (BE = 284.8 eV) was calibrated prior to the analysis of the XPS results. As can be seen in Figure 10, the binding energies of Mn 2p 3/2 and Mn 2p 1/2 have the values of 641.5 ± 0.3 eV and 653.5 ± 0.3 eV, respectively. They coincide with the Mn 2p binding energy of Mn (IV), as reported by Ilton et al. (2016) And the binding energy of the Mn 3s orbital, ΔE (Peak position spacing) is about 4.4 eV. This is consistent with the Mn 2p result, proving that Mn was in the form of Mn (IV) in both the curing system and in the feedstock. Furthermore, the binding energies of Cr 2p 3/2 and Cr 2p 1/2 were found to be 576.8 ± 0.3 eV and 586.7 ± 0.3 eV, respectively. These values were identical to the Cr 2p binding energies of Cr (III), as reported by Zhang et al. (2021). Also, the binding energies of Zn 2p 3/2 and Zn 2p 1/2 were found to be 1,020.8 ± 0.3 eV and 1,043.9 ± 0.3 eV, respectively. These ones were identical to the Zn 2p binding energies of Zn (II) reported by Kabongo et al. (2017). Moreover, the binding energies of Cd 3 days 5/2 and Cd 3 days 3/2 were 405.1 ± 0.3 eV and 412.1 ± 0.3 eV, respectively. They were identical to those of Cd (II), as reported by Dou et al. (1998). Finally, the binding energies of Pb 4f 7/2 and Pb 4f 5/2 were 138.3 ± 0.3 eV and 143.1 ± 0.3 eV, respectively. They were identical to those of Pb (II), as reported by Rondon and Sherwood (1998).

For all of these heavy metal elements, the electronic binding energies were found to be very similar for the raw material, base glass, and glass-ceramic samples. This indicated that the valence states did not change during the whole preparation process. The Pb and Zn elements existed in the form of oxides in the raw materials. They did not participate in the formation of phases in the solidified body and were solidified in the form of a physical coating. The respective electron binding energies were, therefore, almost unchanged during the preparation process. Furthermore, the Cd element was added in the form of a nitrate to the raw material. It was transformed into its oxide form during the high temperature melting process. The electronegativity around the Cd atoms did, thereby, slightly increase, which was also the situation for the electron binding energies in the Cd atoms. Furthermore, the Cr and Mn elements were observed to participate in the formation of the crystalline phases. The strong “oxyphilicity” of Cr lead to the formation of Cr^3+^ ions, and Mn^4+^ ions could easily combine with non-bridging oxygens to form bonds. This resulted in higher electronegativity around the heavy metal ions than what is the case in the raw slag system, and thereby, also lead to lower electron binding energies.

In summary, the solidification of heavy metal elements in glass-ceramics was found to take place in either of two different ways. One was the physical encapsulation, in which heavy metal elements became immobilized in a dense structure formed by a close interlacing of crystalline and amorphous phases in the glass-ceramic. The second type of method was the chemically stabilized solidification, where heavy metal ions became substituted in the crystalline phase, with ions of similar radius size and charge. For instance, Fe and Mn ions could take the place of Al ions in the spinel phase, forming (Mn_0.113_Fe_0.977_Ti_0.91_) [(Ti_0.09_Fe_0.815_Mn_0.095_) O_4_]. Alternatively, heavy metals could also use elements in the solidification system to directly form a stable new phase. For example, Cr and Fe could use Mg in the raw material to form MgFe_0.9_Cr_1.1_O_4_. Thus, the solidification of heavy metal elements in glass-ceramics could take place by a combination of physical encapsulation and chemically stabilizing solidification. As a result, in the present study, the three elements Pb, Cd and Zn were not observed to change significantly during the whole crystallization process. On the other hand, Cr^3+^ was found to promote the enrichment of Mn and Fe in forming a spinel phase, which was generated in the nucleation stage and precipitated as a heterogeneous shaped nucleation core in the crystallization stage. This facilitated the generation and growth of crystals in the crystallization stage and promoted the generation of the main crystalline tremolite phase. It can then be concluded that the microcrystalline phase in the glass-ceramic acted synergistically with the glass phase to solidify the heavy metal ions. That is, the generation of stable crystalline phases, or physically encapsulated heavy metal elements in the crystalline and glass phases, was the result of the joint action of the microcrystalline and glass phases.

## 4 Conclusion

1) Successfully prepared glass-ceramic with the main crystalline phase of diopside using 100% industrial solid waste as raw material. With a high slag elimination capacity, the product performance also reaches the standard of industrial use of plates. Among them, the bending strength of the GC-6 group samples reached 107.35 MPa, Vickers hardness reached 6.67 GPa, density was 3.05 g/cm^3^, water absorption was 0.04%, acid and alkali corrosion resistance of 99.38% and 99.86% respectively.
2) The results achieved by treating harmful heavy metals in waste sludge by glass-ceramic curing are obvious. The curing rate of Cr, Mn, Cd, Pb, and Zn all reached 99.9% for five heavy metal elements. Of them, Cr has the highest curing rate of 99.99%. In contrast to unheated base glass, heavy metal elements in glass-ceramic are more often retained in the curing system in a residue state that can be stabilized in the environment. Moreover, the STIM index of glass-ceramic has been reduced from the level of 3–4 in the base glass state to below 2.
3) The heavy metal elements Cr and Mn are mainly solidified by participating in the formation of crystalline phases, while the three elements Pb, Cd, and Zn are stabilized in the system in the form of physical encapsulation. Using the characteristics of the interlaced distribution of glass and crystal phases in the glass-ceramic system, the two approaches work synergistically to achieve a more excellent stabilized curing effect of heavy metals.

## Data Availability

The original contributions presented in the study are included in the article/Supplementary Material, further inquiries can be directed to the corresponding author.
